# An Overview of Systematic Reviews of Danhong Injection for Ischemic Stroke

**DOI:** 10.1155/2016/8949835

**Published:** 2016-07-31

**Authors:** Hui Wang, Shutian Ren, Chunxiang Liu, Xiaoxia Zhang

**Affiliations:** ^1^Tianjin Institute for Clinical Evaluation of Traditional Chinese Medicine, Tianjin University of Traditional Chinese Medicine, Tianjin 300193, China; ^2^Tianjin State Key Laboratory of Modern Chinese Medicine, Tianjin University of Traditional Chinese Medicine, Tianjin 300193, China; ^3^Massage Department, Tianjin Nankai Hospital, Tianjin 300100, China

## Abstract

*Objective.* This overview is to evaluate the current evidence from systematic reviews (SRs) of Danhong injection (DHI) for ischemic stroke (IS).* Methods.* SRs of randomized controlled trials (RCTs) concerning DHI and IS were searched in six databases without language restrictions until September 2015. Assessment of multiple systematic reviews (AMSTAR) was used to evaluate the methodological quality of all included SRs.* Result.* A total of 8 articles were included. After the administration of DHI, clinical efficiency and neurological deficits score have marked advantages over those of the control group. However, the overall poor quality of meta-analysis and original studies affected the reliability of the results. Evaluation of methodological quality found that no one paper meets the requirements of all 11 items. The main flaws of the methodology quality included the following: not providing “a priori” design and reasonable objectives, duplicate study selection not given enough attention, performing an incomprehensive literature search, not paying attention to publication bias and other bias reports, characteristics of included studies not provided in detail, and ignoring clinical heterogeneity when performing meta-analyses.* Conclusion.* The current published SRs suggest DHI appears to be a safe and effective way for IS treatment in general. However, it lacks a high quality systematic evaluation and analysis. The quality of SRs should be improved. Further large sample-size and well-designed RCTs are needed.

## 1. Introduction

Ischemic stroke (IS), also known as cerebral infarction stroke, is a common cardiovascular disease; it refers to the brain blood supply obstacles, ischemia, and hypoxia that cause brain tissue necrosis osteomalacia. It is a clinical frequent and common disease [1]. This disease is common worldwide and there are more than 680000 adult cases of ischemic stroke annually in the United States [2]. 700 million people are suffering from stroke in China now [3]. There are about 200 million patients with cerebral stroke each year; 70%–80% of patients cannot live independently. Morbidity and mortality in acute stroke patients significantly increased with age increase, which threatens the health of human beings [4]. IS causes a lot of damage to body and mind and seriously affects the quality of life [5]. The prevention and treatment of IS are important to improve the health of people.

Evidence based medicine has proved that thrombolytic therapy is the most effective method for treating acute ischemic stroke. However, only a few people can get thrombolytic therapy. Wang et al. reported the situation of intravenous recombinant tissue plasminogen activator (rt-PA) thrombolytic therapy in China; the results show that thrombolytic rate of patients with IS was only 1.3% [6]. As seen above, thrombolytic therapy cannot be widely used in clinical practice, and then the secondary prevention of ischemic stroke is particularly important. Methods such as acupuncture treatment, integrative medicine therapy [7], and traditional Chinese medicine (TCM) have been wildly used. Among these treatments, TCM injection is a good way for IS because of its convenience, remarkable curative effect, less adverse reaction, and low cost.

Danhong injection (DHI) is made of the extraction from Danshen (Radix Salviae Miltiorrhizae) and Honghua (Flos Carthami). The ingredients of DHI are mainly tanshinone, salvianolic acid, safflower yellow pigment, safflower phenolic glycosides, and so forth [8]. DHI has an effect of dispersing blood stasis and dredging collateral, protecting vascular endothelium, promoting angiogenesis, inhibiting platelet aggregation and anticlotting, and so on. It is mainly used for cerebral infarction and other cardiovascular/cerebrovascular diseases in clinical practice [9].

Many clinical trials of DHI for IS have been done and mainly published in Chinese journals. Furthermore, there were also some SRs/meta-analyses about DHI for ischemic stroke published. But the methodological quality is uneven. High quality SRs and meta-analysis are the main way to ensure the best available evidence. On the contrary, it will be misleading to clinical decision-making. In view of this, this paper aimed to summarize and critically appraise the evidence of relevant SRs and use AMSTAR scale to evaluate the methodological quality in order to understand the current situation and problems of SRs/meta-analyses about DHI for ischemic stroke.

## 2. Method

### 2.1. Eligibility Criteria

SRs have to be concerned specifically with the effectiveness of DHI on ischemic stroke. There were no limitations to the publishing date, language, and outcome measures. SRs evaluating DHI together with other Chinese herbal medicines and without separate evaluation of the individual drug were excluded. Reviews, comments, conference abstracts, research proposal, and overviews without systematic methods section were excluded.

### 2.2. Search Strategy

A comprehensive computer literature search was conducted to find relevant published articles on this topic, using databases including the Cochrane Library, PubMed, China National Knowledge Infrastructure (CNKI), Wanfang data, and VIP Database and the Chinese Biomedical Literature Database (Sinomed). We used a search algorithm based on a combination of the terms (“Danhong” OR “Dan hong”) AND (“Systematic review” OR “meta-analysis”) AND (“Cerebral Infraction” OR “Ischemic Stroke”). Chinese databases were searched using the abovementioned search terms in Chinese accordingly. The search was updated until September 2015. We also reviewed the reference lists of identified publications for additional pertinent reviews. No language restrictions were imposed.

### 2.3. Data Extraction

We extracted data on SRs (the first author's last name, year of publication, number of papers included in the review, methodological details, and intervention and outcome measured). Two reviewers (HW, STR) independently examined the titles and abstracts of the literature for inclusion, based on the selection criteria outlined above. The full texts of articles were retrieved if there was any doubt whether an article should be included or not. Inconsistencies were solved through discussion.

### 2.4. Quality Assessment

All eligible systematic reviews were assessed using a measurement tool for the assessment of multiple systematic reviews (AMSTAR). AMSTAR is a recognized scale that can evaluate methodological quality of SRs and meta-analysis, which consists of 11-item scale, is highly recommended scale for the evaluation of the methodological quality [10]. The first choice has been recommended to some organizations [11]. AMSTAR has been internally and externally validated and has been found to have good reliability [10]. The 11 items were assessed for each review and the total number of positive answers for each was documented. For each item, there are four answers for choosing: “cannot answer,” “yes,” “no,” and “not applicable.” Two authors (H. Wang, S. Ren) independently performed quality assessment. Disagreements were resolved by discussion or consultation with a third individual (C. Liu).

## 3. Results

### 3.1. Literature Search Results

**Figure 1:**
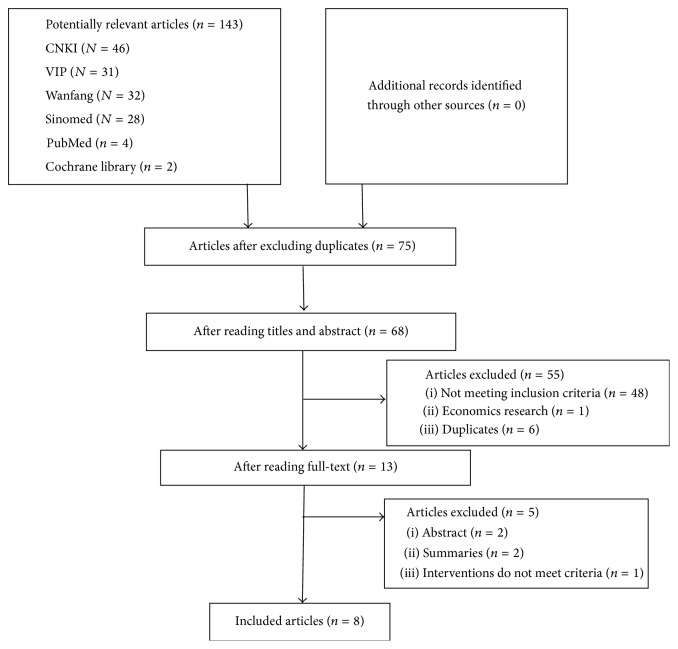
PRISMA 2009 flow diagram [12].

Figure 1 shows a flow diagram describing the study selection process. The initial search yielded 143 research reports, of which 75 were excluded for having the same title or authors. 55 studies were excluded after reading titles and abstracts (not including SRs, inappropriate population, inappropriate intervention, or not meeting inclusion criteria). Five were excluded after reading full contexts. A total of 8 SRs were included in this overview.

### 3.2. Characteristics of SRs

All eight systematic reviews were published between 2009 and 2014. SRs were based on 9 to 45 primary studies with a total of 16469 participants. Characteristics of included studies were summarized in Table 1. Trials including adult patients with IS were eligible, in which five studies include acute ischemic stroke. Treatment group uses DHI, 20–40 mL, 1 time/d, or combined conventional therapy; the control group mainly uses Compound Danshen Injection, routine treatment, and other Chinese and Western medicines such as dextran injection and Venoruton injection. The treatment courses of original studies ranged from 7 to 30 days. The main outcomes were clinical efficacy of neurological deficit and improvement of neurological function. Only one study reported the deterioration and mortality as the primary endpoint. Seven studies reported adverse event (AE). The Jadad scores were used in 5 systematic reviews, quality assessments according to Cochrane Handbook for Systematic Reviews were used in another 3 studies, and most of the primary studies were of poor quality.

### 3.3. Methodological Quality Assessment

According to the AMSTAR scores, the quality of these reviews was varied. The results are shown in Table 2. The number of reviews satisfying the criteria for individual items varied widely.

Two items were satisfied by over 75% of the SRs, namely, Item 7 (7 (87.5) SRs assessed the scientific quality of the included studies and 5 SRs used Jadad scale score, while other 3 SRs used quality assessment on Cochrane Systematic Review's Handbook, in which 87.5 percent give a score or rank in detail) and Item 10 (7 SRs give an assessment of publication bias, funnel plot analysis suggested publication bias was small in some study [15, 16, 18–20], and greater possibility of publication bias was prompted by some other studies [13, 14]).

In contrast, nine items accounted for the major methodological limitations: For the included 8 systematic reviews, in Item 1, none of them provide “a priori” design, all of eight SRs were not registered, and 4 of the literatures did not report exclusion criteria; and in Item 2, three (37.5%) of the studies showed there were duplicate study selection and data extraction. Data were independently extracted by two researchers, and disagreements were resolved by discussion. In Item 3, in the aspect of the comprehensive literature search strategy, seven (87.5%) studies provided search terms, of which only two (25%) provide search strategy and four (50%) provide the literature supplemented retrieval. In Item 4, language bias existed in all 8 articles and none of the authors stated whether they included grey literature. Three of them clearly stated retrieval domestic literature, one did not explicitly described, but only retrieve Chinese database. In Item 5, all the reviews provided a list of included studies, while a list of excluded studies was not reported. In Item 6, 4 articles provided detailed characteristics of the included studies, with 2 of them in a form of table. In Item 8, scientific quality of 3 included studies was used appropriately in formulating conclusions; in Item 9, 3 SRs adopted appropriate methods of meta-analysis; in Item 11, the interests conflict statement was not met by any of the SRs but one.

### 3.4. Primary Outcomes

#### 3.4.1. Clinical Efficiency

All eight SRs indicated that, compared with control group and treatment group, the difference was statistically significant. DHI or DHI plus RT can improve the neurological deficit and improve clinical efficiency in patients with ischemic stroke [13–20]. The results are shown in Table 3.

#### 3.4.2. Neurological Deficits Score

Five SRs yielded a positive result that suggested DHI or DHI plus RT did significantly differ from control group in improving the degree of neurological deficit [13, 14, 17, 19, 20]. WMD of effect sizes range from −3.77 [95% CI, −2.63 to −4.91] to −4.59 [99% CI, −6.84 to −2.35] as shown in Table 4.

#### 3.4.3. Deterioration and Mortality

Peng's et al. study [19] is the only article that took the deterioration and mortality as the main indicator (10 trials). A total of 800 cases were included. The observation group received DHI and control group received Danshen injection. Each group had 400 cases. Deterioration rate and fatality rates results showed that DHI can significantly decrease the retreatment rate due to deterioration. 11 patients in the treatment group and 34 patients in control group had clinical deterioration or died (OR = 0.33, 95% CI, 0.17–0.63, *P* = 0.001).

### 3.5. Adverse Effects

In five reviews addressing the adverse effects of DHI (Table 5), there are three types of AE occurrence: “not reported,” “no AE occurred,” and “AE occurred.” AE included dizziness, rash, fever, skin flushing, and gastrointestinal symptoms. Result of AE description consistent with AE was described in the specification. All in all, this systematic review indicated that DHI had low AE occurrence.

## 4. Discussion

This overview indicated that SRs of DHI for IS have emerged between 2009 and 2014, suggesting that the interests of the public and the medical profession in the use of DHI for healthcare have grown considerably in recent years.

### 4.1. Methodological Qualities of SRs

The AMSTAR scale was selected to assess various aspects of the methodological quality of SRs. This paper included eight SRs. However, the methodological quality assessment of the included SRs reveals there are common areas for improvement.

(a) SRs did not provide a priori design and reasonable argumentation, only indicating the inclusion criteria and exclusion criteria in the text may cause duplication of SRs published.

(b) Seven studies (87.5%) concerned the extraction of repeatability, while duplicate study selection did not give enough attention. Comprehensive data collection is a fundamental principle and therefore needs to focus on study selection and also requires two reviewers independently and verifying consistency.

(c) The database resource retrieval, retrieval of years, search terms, and search strategies are very critical information in SRs; however, we found that only two SRs (25%) provide a search strategy; the rest were not mentioned. There will be risk of bias because of incomprehensive literature searches. There were 4 reviews that only searched Chinese databases. None of the authors stated whether they included grey literature. As we know, SRs should search not only all relevant published literature both at home and abroad, but also gray literature without limit of the language, so as to avoid publication bias and language bias and so on.

(d) The quality level of the original studies directly reflects the strength of evidence of SRs. However, most of the original studies are of lower quality, and this thus affects the final data integration authenticity and reliability of the results.

(e) For meta-analysis, researchers only consider the statistical heterogeneity when combining the findings of studies and formulating conclusions, and it is difficult for authors to determine the clinical and methodological heterogeneity because characteristics of the included studies were not provided in detail. Moreover, DHI is traditional Chinese medicine injection; it will be affected by the dose, course of treatment, and the control group interventions varied; blind merger is also not feasible.

(f) There is only one reported source of funding; the rest were not reported, because the current domestic journals did not carry out the requirements that relate to this phenomenon.

### 4.2. Summary of Evidence

#### 4.2.1. Statement of Main Findings

The purpose of the present overview was to critically evaluate the evidence from SRs and to provide a rigorous and objective summary concerning the effectiveness of DHI in the treatment of IS. Overall analysis suggests that, (1) for clinical efficiency, there existed consistent evidence that DHI had a more favorable effect than control group when used alone or DHI was added to another conventional intervention; (2) for neurological deficits score, consistent evidence found that DHI provides better functional improvement when compared with control group; (3) one study took deterioration and mortality as the main indicator; the results also suggest DHI produces a clinically significant reduction in deterioration and mortality when compared to CDI. Nevertheless, because the study course is short, secondary outcomes are mainly used as outcome measurement and without longtime follow-up. As we know, for acute stroke patients, reduced mortality and morbidity, improved quality of life, and prolonged survival time are the focus of treatment. But only one study mentioned fatality reports, also there were no reports of indicators such as quality of life, and long-term effects on the evaluation need further observation.

#### 4.2.2. Safety

The result showed that adverse reactions of DHI were mild and consistent with adverse reactions described in the specification. It is suggested that the safety is better. In addition, another literature analysis with 436 clinical trials reports similar conclusions [21]. AE of DHI is mostly capable of self-limiting. However, it should be noted that some studies did not report the result of AE or detailed description as shown in Table 5. Two reasons may cause this situation: first, original research did not report relevant information; secondly, the original research has reported it, but the SRs did not extract relevant data. Security is a vital part in clinical research; we recommend authors pay enough attention.

### 4.3. Limitations

We have made efforts to minimize the risk of bias in every step of this overview. For instance, for literature identification, we used systematic, comprehensive, and independent search strategies over a wide range of English and Chinese electronic databases, without restriction of language and year of publication. However, DHI belongs to Chinese traditional medicine prescription and is only available in the domestic market. By searching the literature, the authors also found all the studies were for the Chinese people and published in China. Thus, there was publication bias. The low quality of the original studies and insufficient details to display for characteristics of included studies affected the reliability of the results. The improper use of meta-analysis will exaggerate bias and draw incorrect conclusions. Moreover, in practical application, we found that there is a certain ambiguity of evaluation items, although two reviewers independently assessed AMSTAR score; inconsistencies were solved through discussion, but still the problem of subjective judgment may exist.

### 4.4. Implication of Practice

The results suggest that the methodological quality of SRs should be improved. We recommend that researchers engaged in systematic reviews should receive relevant training. Method of literature search, quality assessment, results analysis, report writing, and so forth should be given more attention. The accuracy and repeatability of findings can be ensured through guidelines such as the Consolidated Standards of Reporting Trials (CONSORT) statement [22] and CONSORT for TCM [23] when designing and reporting RCTs.

## 5. Conclusions

Based on eight systematic reviews, DHI for ischemic stroke is effective in clinical efficiency and functional improvement. DHI seems generally safe for clinical application. However, poor quality of systematic reviews/meta-analyses affected reliability of current evidence. Further large sample-size and well-designed RCTs are crucial for confirming this conclusion. In addition, there is a need for higher quality of SRs.

## Figures and Tables

**Table 1:** Characteristics of systematic reviews.

Author, year	Electronic databases (year)	Number of studies included (number of study population)	Intervention (participants)	Comparison interventions (participants)	Course (d)	Main outcome	Types of participants	Quality assessment	Quality of primary studies
Hu et al., 2009 [13]	CNKI, Wanfang, VIP (1994–2008)	12 (950)	DHI + RT (477)	CDI, XSTI, VI, BC + RT (473)	14–21 d	①②	AIS	Jadad	Poor

Xu et al., 2010 [14]	CNKI, Wanfang, VIP, CBM (NA)	9 (996)	DHI	XDI, CXQI HI, RT, DI, CDI, XSTI	14–30 d	①②	AIS	Jadad	Poor

Xiong et al., 2014 [15]	CBM, FMJS, CNKI, Wanfang, VIP, duxiu (2009–2014)	21 (2725)	DHI	NA	NA	①	IS	Jadad	Poor

Ye et al., 2010 [16]	CNKI, PubMed (2004–2009)	45 (4027)	DHI	CDI, RT, VI, XSTI, CXQI, and so forth	7–30 d	①	IS	Jadad	Poor

Yang and Zeng, 2012 [17]	CNKI, Wanfang (2006–2010)	27 (2583)	DHI + RT	DSI, CDI + RT	NA	①②	IS	Cochrane Handbook	B Grade

Liu et al., 2014 [18]	CNKI, Wanfang (2004–2014)	9 (885)	DHI + RT	RT	7–14 d	①	AIS	Cochrane Handbook	B or C Grade

Peng et al., 2010 [19]	Medline, Embase, CBM, CNKI, VIP, Wanfang (2000–2010)	29 (3191)	DHI	CDI	14–30 d	①②③	AIS	Cochrane Handbook	B Grade

Ma et al., 2012 [20]	CBM, VIP, Medline, Cochrane (up to 2011)	14 (1112)	DHI + RT	RT	14–21 d	①②	AIS	Jadad	Poor

DHI, Danhong injection; RT, routine therapy; CDI, Compound Danshen Injection; XSTI, Xuesetong injection; VI, Venoruton injection; BC, black control; XDI, Xiangdan injection; CXQI, Chuanxiongqin injection; HI, hydroxyethyrutin injection; DI, Dextran injection; NA, not available; DSI, Danshen injection; AIS, acute ischemic stroke; IS, ischemic stroke; FMJS, foreign medical journal full-text service.

① Clinical efficiency; ② neurological deficits score; ③ deterioration and mortality.

**Table 2:** Methodological quality assessment of systematic reviews.

AMSTAR scale	Yes		No		Cannot answer or not applicable	
	*n*	%	*n*	%	*n*	%
1						
Research problem	0	0	8	100	0	0
Inclusion criteria	8	100	0	0	0	0
Exclusion criteria	4	50	4	50	0	0

2	3	37.5	5	63.5	0	0

3						
Electronic sources	8	100	0	0	0	0
Search terms	7	87.5	1	12.5	0	0
Search strategy	2	25	6	75	0	0
Supplemented retrieval	4	50	4	50	0	0

4						
Grey literature	0	0	8	100	0	0
Language restrictions	4	50	4	50	0	0

5	0	0	8	100	0	0

6						
Characteristics	4	50	4	50	0	0
Table	2	25	6	75	0	0

7	7	87.5	1	12.5	0	0

8	3	37.5	5	63.5	0	0

9	3	37.5	5	63.5	0	0

10	7	87.5	1	12.5	0	0

11	1	12.5	0	0	7	87.5

*Note.* (1) Was an “*a priori*” design provided? (2) Were there duplicate study selection and data extraction? (3) Was a comprehensive literature search performed? (4) Was the status of publication (i.e., grey literature) used as an inclusion criterion? (5) Was a list of studies (included and excluded) provided? (6) Were the characteristics of the included studies provided? (7) Was the scientific quality of the included studies assessed and documented? (8) Was the scientific quality of the included studies used appropriately in formulating conclusions? (9) Were the methods used to combine the findings of studies appropriate? (10) Was the likelihood of publication bias assessed? (11) Was the conflict of interests stated?

**Table 3:** Summary of positive results with meta-analysis and clinical efficiency.

Author, year	Number of studies included (population)	Main outcome
Hu et al., 2009 [13]	11 (858)	OR = 3.30 [95% CI, 2.22–4.91]
Xu et al., 2010 [14]	9 (996)	OR = 3.15 [95% CI, 2.22–4.48]
Xiong et al., 2014 [15]	21 (2822)	OR = 3.59 [95% CI, 2.87–4.48]
Ye et al., 2010 [16]	45 (4027)	OR = 3.14 [95% CI, 2.65–3.73]
Yang and Zeng, 2012 [17]	27 (2583)	RR = 1.21 [95% CI, 1.17–1.26]
Liu et al., 2014 [18]	9 (885)	OR = 3.69 [95% CI, 2.52–5.42]
Peng et al., 2010 [19]	27 (3010)	OR = 3.83 [95% CI, 3.10–4.73]
Ma et al., 2012 [20]	13 (1072)	RR = 1.22 [99% CI, 1.14–1.31]

**Table 4:** Summary of positive results with meta-analysis and neurological deficits score.

Author, year	Number of studies included (population)	Main outcome
Hu et al., 2009 [13]	9 (740)	WMD = −4.04 [95% CI, −4.85 to −3.23]
Xu et al., 2010 [14]	2 (136)	WMD = −4.25 [95% CI, −6.63 to −1.86]
Yang and Zeng, 2012 [17]	16 (1641)	WMD = −4.55 [95% CI, −5.46 to −3.63]
Peng et al., 2010 [19]	13 (1356)	WMD = −3.77 [95% CI, −2.63 to −4.91]
Ma et al., 2012 [20]	10 (748)	WMD = −4.59 [99% CI, −6.84 to −2.35]

**Table 5:** Summary of AE.

Author, year	Number of trails (participants)	AE not reported	No AE occurred	AE occurred	AE description (number of cases)
Hu et al., 2009 [13]	12 (950)	3	5	4	T: dizziness (2), rash (2); C: allergies (3); rash (1)

Xu et al., 2010 [14]	9 (996)	3	3	3	T: headache (1), skin flushing (1), gastrointestinal symptoms (3); unstable blood pressure (2)

Xiong et al., 2014 [15]	21 (2725)	21	0	0	/

Ye et al., 2010 [16]	45 (4027)	23	NA	NA	T: headache (5); C: rash (2), pruritus (2), injection site bruising (1), bleeding gums (1)

Yang and Zeng, 2012 [17]	27 (2583)	8	17	2	T: rash (2), fever (2)

Liu et al., 2014 [18]	9 (885)	NA	NA	NA	NA

Peng et al., 2010 [19]	29 (3191)	NA	NA	NA	T: gastrointestinal symptoms (1), fever (2), dizziness (3), rash (2); C: rash (1), fever (2)

Ma et al., 2012 [20]	14 (1112)	6	5	3	Ecchymosis, T/C unknown

T, treatment group; C, control group; NA, not available.
